# Evaluation of the Nutritional Quality of Chinese Processed Meat Products: Comparison of Two Nutrient Profile Models

**DOI:** 10.3390/nu16050578

**Published:** 2024-02-20

**Authors:** Xin Ding, Wanting Lv, Yang Liu, Ying Lu, Yajun Liu, Hanning Li, Beilei Cai, Junhua Han, Yuexin Yang, Chao Gao, Zhu Wang

**Affiliations:** 1 National Institute for Nutrition and Health, Chinese Center for Disease Control and Prevention, Beijing 100050, China; 2021021102@qdu.edu.cn (X.D.);; 2 Institute of Nutrition and Health, School of Public Health, Qingdao University, Qingdao 266071, China; 3 Chinese Nutrition Society, Beijing 100053, China; 4 Key Laboratory of Trace Element Nutrition of National Health Commission, Beijing 100050, China

**Keywords:** nutrient content, processed meat products, nutrient profiling, front-of-pack labeling, food policy

## Abstract

Processed meat products are one of the most consumed pre-packaged foods in China. They are also group-1 carcinogens, whose consumption has proved to be positively associated with the risk of noncommunicable diseases (NCDs). The purpose of this study is to analyze the nutrient content on the food label of processed meat products based on the China Standardized Database for the Composition of Pre-packaged Food and the National Open Database of the UK and France. The Chilean front-of-pack warning label (FOPWL) and the Chinese Healthier Choice Logo were used to compare the nutrient content of processed meat products from the three countries. It was found that cured meat products have the highest median energy (483 kcal/100 g), total fat content (38.7 g/100 g), and sodium content (2076 mg/100 g) and dried meat products have the highest median protein content (30.2 g/100 g) and carbohydrate content (38.2 g/100 g). In addition, there were significant differences in energy content and contents of total fat, protein, and carbohydrate across different products of the three countries (*p* < 0.001). A large number of processed meat products currently collected did not meet the criteria of the Chilean FOPWL and the Chinese Healthier Choice Logo. This study provided information on the healthiness of Chinese processed meat products and provided data for improving food formulations for different categories of processed meat products.

## 1. Introduction

Globally, unhealthy diets have caused 11 million deaths and 255 million disability-adjusted life years (DALYs) [1]. The main drivers of overweight, obesity, and diet-related noncommunicable diseases (NCDs) are excessive intakes of critical nutrients (CN), such as sodium [2,3,4], sugars [5,6], and saturated fats [7,8], which are found in high concentrations in pre-packaged foods [9,10]. Processed meat products are pre-packaged foods commonly consumed in China [11,12]. In developed countries, it is estimated that sodium intake from meat and meat products contributes approximately 20% of total daily sodium intake in developed countries [13].

Processed meat products have been classified as carcinogens (group 1) [14]. The consumption of processed meat products has been demonstrated to have detrimental correlations with colorectal and stomach cancer [15,16], cardiovascular disease [17], stroke [18], and type 2 diabetes [19]. Furthermore, compared to food categories such as sauces and instant noodles, the reformulation of processed meat products is less likely to achieve its nutritional goals, i.e., sodium reduction [20], which may be due to its essential functions in providing flavors, texture, and shelf life [13].

Nutrient profiling (NP) is the tool of classifying or ranking foods according to their nutritional composition, which informs the formulation and application of strategies for the prevention and control of obesity and overweight, such as front-of-pack labeling (FOPL) and food reformulation [21]. Chile, Uruguay, and Mexico adopted front-of-pack warning labels (FOPWLs) [22]; France, Spain, and Germany adopted Nutri-Score [23,24]; China [25], Thailand, and Malaysia used endorsement logos (e.g., Choices) [24]. The calculation methods behind the various NP models are different. For example, the implementation of FOPWL is backed by a binary system in which products exceeding thresholds must carry the label; Nutri-Score allows for graded labeling (high–medium–low), informed by different cut-offs for different nutrients; products are only eligible to carry the endorsement logo if nutrition criteria are met [26].

Previous studies have analyzed the sodium distribution among China’s processed meat. However, there has been a lack of analysis of the nutrient conditions of energy, protein content, fat content, carbohydrate content, and sodium content on the food labels of various processed meat product categories. Therefore, the purpose of this study is to meticulously classify and analyze processed meat products available in the China Standardized Database for the Composition of Pre-packaged Food. In addition, the nutrient content of processed meat products in the National Open Database from the UK and France has also been analyzed. We used the Chilean FOPWL and the Chinese Healthcare Choice Logo to compare the difference in nutrient content among the three countries. This study provides data for improving food formulations for different categories of processed meat products and provides evidence of best practices to scientifically guide meat intake in China.

## 2. Materials and Methods

### 2.1. Data Collection

#### 2.1.1. Sample Collection of Chinese Processed Meat Products

The National Institute for Nutrition and Health, Chinese Center for Disease Control and Prevention (NINH, China CDC), has established the China Standardized Database for the Composition of Pre-Packaged Food following the relevant provisions in the Standard on Nutrition Labelling of Pre-Packaged Foods (GB 28050-2011) [27]. The principles of collecting pre-packaged foods include: the pre-packaged food is within the shelf life; the food information on the pre-packaged food is clear and inclusive (e.g., brand, product name, nutrition information panels (NIPs), and ingredients); the sum of energy converted from fat, carbohydrates, and protein on the label does not exceed the energy value on the label; nutrient content in products is standardized to volume units per 100 g or 100 mL.

The data for this study include the nutrition label information of processed meat products in 20 provinces of China collected from 2017 to 2022 from the China Standardized Database for the Composition of Pre-Packaged Food. The collection provinces covered seven major geographical regions of China, which were divided by Chinese population density and economic correlation, including Guangdong Province, Jiangsu Province, Shandong Province, Zhejiang Province, Henan Province, Hebei Province, Beijing City, Chongqing City, Inner Mongolia Autonomous Region, Xizang Autonomous Region, Qinghai Province Hainan Province, Gansu Province, Jilin Province, Heilongjiang Province, Xinjiang Uygur Autonomous Region, and Guizhou Province.

#### 2.1.2. Sample Collection in the UK and France

The data of processed meat products come from the following sources: UK—McCance and Widdowson’s Comprehensive Food Ingredient Dataset (CoFID) (2021) [28]; France—Oqali database [29].

### 2.2. Data Classification

**Figure 1:**
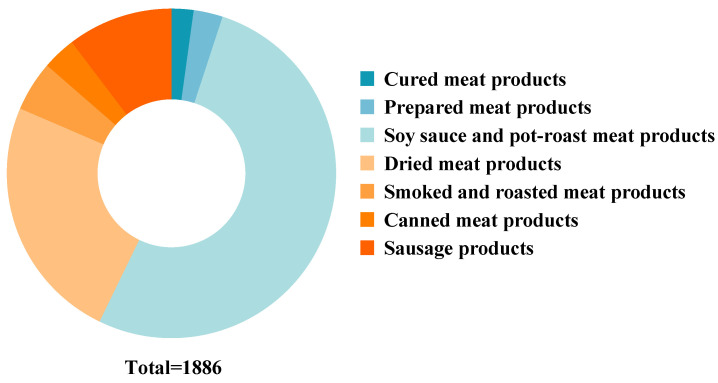
Distribution of Chinese processed meat products.

Chinese processed meat products were categorized according to Chinese food standards (including national standards, group standards, and industrial standards), the characteristics, processing technologies, formulation of processed meat products, and Chinese dietary habits (Supplementary Table S1). The specific number and distribution of processed meat products are shown in Figure 1.

In light of the original food classification in the UK and France, the researchers cross-referenced the processed meat product categories of China and decided on the relevant data to be included in the study (Supplementary Figure S1).

### 2.3. Data Inclusion and Exclusion Criteria

Processed meat products with complete NIPs and ingredients were included. Processed meat products with incorrect or incomplete nutrition information and products that were missing ingredient information or could not be classified into target categories were excluded from the analysis.

### 2.4. Nutrient Profiling Models

The Chilean FOPWL and the Chinese Healthier Choice Logo were used to cross-compare the nutrient content of processed meat products in China, the UK, and France. China Consumer Surveys on FOPL [30,31,32] and other studies on the view of stakeholders on the FOPL policy in the Chinese context [33,34] indicated that Chinese residents and stakeholders tended to prefer interpretive FOPL. Furthermore, due to the limitations of mandated labeling contents of Chinese food labeling standards, summary-based graded NP models that evaluate overall nutritional quality of foods, such as Nutri-Score, cannot be used in this study to evaluate the nutrient content of products. Table 1 summarizes the characteristics and thresholds of the Chilean FOPWL and the Chinese Healthier Choice Logo.

Chilean FOPWL: In a staggered way, thresholds become increasingly stricter over 3 phases. The third-phase threshold was implemented in 2019. When the content of energy, total sugar, saturated fat, and sodium in products exceed the threshold, the front of the packaging must be labeled with a black octagonal FOPWL [35,36].

Chinese Healthier Choice Logo: The Chinese Nutrition Society released China’s first interactive FOPL system in 2018 and implemented it in 2019. This FOPL aims to strengthen health guidance for research and production of pre-packaged foods. It divides products into 10 food categories and 31 subcategories. Products meeting thresholds of total fat, saturated fat, total sugar, added sugar, and sodium are labeled with a “Healthy Choice” FOPL symbol [25].

### 2.5. Data Analysis

The median and 25th and 75th percentiles were used to analyze the distribution of processed meat products by each nutrient and the nutrient reference value (NRV). The Kruskal–Wallis H test was used to determine differences in nutrient content of processed meat products from different categories in China. A *p* value of <0.05 was considered significant. The percentage of processed meat products in China, France, and the UK by category that met the criteria for each nutrient were compared under the Chilean FOPWL and the Chinese Healthier Choice Logo. The analyses were conducted using IBM SPSS V.26.0.

## 3. Results

### 3.1. The Nutrient Content for Chinese Processed Meat Products

**Figure 2:**
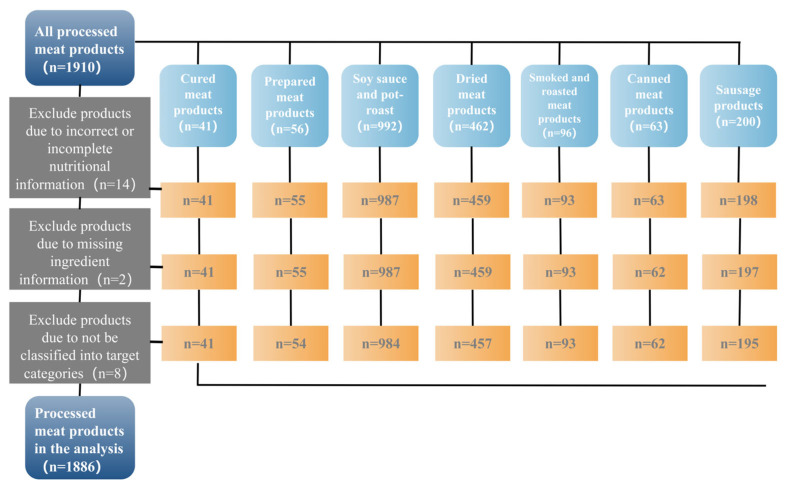
Chinese processed meat products selection process.

From a total of 1910 processed meat products that were collected from China, 14 were excluded due to incorrect or incomplete nutrition information, 2 were excluded due to missing ingredient information, and 8 were excluded due to inability to classify. Finally, this study included a total of 1886 products (Figure 2). Chinese processed meat products were classified into seven categories (Figure 1, Table 1).

Table 2 and Table 3 display the nutrient distribution and NRV of Chinese processed meat products. Cured meat products had the highest median energy (483 kcal/100 g), total fat content (38.7 g/100 g), and sodium content (2076 mg/100 g); the median NRV was 24.2%, 64.5%, and 103.8%, respectively. Dried meat products had the highest median protein content (30.2 g/100 g) and carbohydrate content (38.2 g/100 g); the median NRV was 50.3% and 12.7%, respectively. Prepared meat products had the lowest median energy (135 kcal/100 g), total fat content (7.6 g/100 g), and sodium content (648 mg/100 g); the median NRV was 6.8%, 12.7%, and 32.4%, respectively. Canned meat products had the lowest median protein content (11.2 g/100 g), with a median NRV of 18.7%, and soy sauce and pot-roast meat products had the lowest median carbohydrate content (2.8 g/100 g), with a median NRV wofas 0.9%. Statistically significant differences were observed for energy content, protein content, total fat content, carbohydrate content, and sodium content of processed meat products across categories (*p* < 0.001).

### 3.2. Chinese Processed Meat Products Compared with Selected Processed Meat Products in the UK and France

A total of 44 processed meat products were selected for final analysis from 528 products in the UK, and a total of 1113 processed meat products were selected for final analysis from 1951 products in France (Supplementary Figure S1).

**Figure 3:**
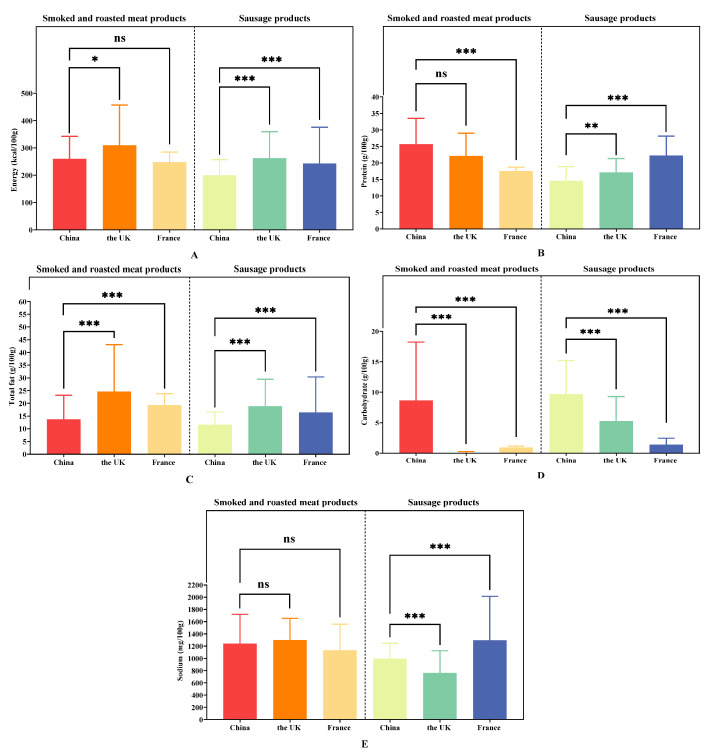
Chinese processed meat products compared with selected processed meat products in the UK and France. (**A**) comparison of energy; (**B**) comparison of protein content; (**C**) comparison of total fat content; (**D**) comparison of carbohydrate content; (**E**) comparison of sodium content. The “*” means *p* < 0.05. The “**” means *p* < 0.01. The “***” means *p* < 0.001. The “ns” means no significance.

From a total of 1157 products that were selected from the UK and France, 1043 were sausage products, and 111 were smoked and roasted meat products. A total of 195 sausage products and 93 smoked and roasted meat products in China were compared with products from the UK and France. For smoked and roasted meat products, the median energy and total fat content of Chinese products were lower than those of the UK and France (energy: 236 kcal/100 g vs. 287 kcal/100 g vs. 253 kcal/100 g, *p* = 0.0046; total fat: 10.2 g/100 g vs. 21.6 g/100 g vs. 20.0 g/100 g, *p* < 0.001); the median protein content and carbohydrate content of Chinese products were higher than those of the UK and France (protein: 26.0 g/100 g vs. 23.8 g/100 g vs. 17.4 g/100 g, *p* < 0.001; carbohydrate: 4.8 g/100 g vs. 0.0 g/100 g vs. 0.8 g/100 g, *p* < 0.001), and products in the UK had the highest median sodium content (1368 mg/100 g vs. 11,500 mg/100 g vs. 1000 mg/100 g, *p* = 0.1433). For sausage products, the median energy, protein content, and total fat content of Chinese products were lower than those of the UK and France (energy: 187 kcal/100 g vs. 267 kcal/100 g vs. 230 kcal/100 g, *p* < 0.001; protein: 13.0 g/100 g vs. 15.8 g/100 g vs. 21.0 g/100 g, *p* < 0.001; total fat: 10.9 g/100 g vs. 19.5 g/100 g vs. 12.5 g/100 g, *p* < 0.001); the median carbohydrate content and sodium content of Chinese products were higher than those of the UK and France (carbohydrate: 9.2 g/100 g vs. 7.2 g/100 g vs. 1.0 g/100 g, *p* < 0.001; sodium: 980 mg/100 g vs. 630 mg/100 g vs. 960 mg/100 g, *p* < 0.001) (Table 2 and Table 4 and Figure 3).

### 3.3. Nutritional Quality of Processed Meat Products in China, the UK, and France under Different NP Models

**Figure 4:**
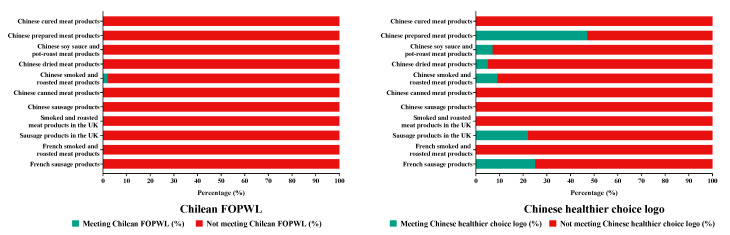
Nutritional quality of processed meat products in China, the UK, and France under the Chilean FOPWL and the Chinese Healthier Choice Logo.

It can be seen from Figure 4 that the ability of the two models to identify processed meat products in China, the UK, and France containing excessive amounts of CNs varied considerably between NP models. The Chilean FOPWL classified only 1.1% of Chinese smoked and roasted meat products as compliant, and all products in other categories did not meet the criteria of this model. The proportion of Chinese processed meat products that did not meet the Chinese Healthier Choice Logo criteria ranged from 53.7% to 100.0%, and under the same criteria of the model, the proportion of processed meat products in the UK was 78.3% to 100%, while the proportion of French processed meat products was 75.7% to 100%.

Based on the criteria of the Chilean FOPWL, a large proportion of products in categories of Chinese sausage products (4.9%), Chinese canned meat products (5.3%), and smoked and roasted meat products in the UK (38.1%) did not meet the threshold of energy; the proportion of other categories of processed meat products in China, the UK, and France that met the threshold of energy was relatively high, ranging from 58.1% to 98.1%. A total of 16.7% of Chinese prepared meat products met the threshold for sodium; the proportion of other categories of processed meat products in China, the UK, and France that met the threshold for sodium was relatively low, ranging from 0.0% to 4.3%. The proportion of processed meat products in the UK and France that met the threshold for saturated fat was 6.7% to 45.7%. About 100% of processed meat products in the UK and France met the threshold for total sugar (Supplementary Table S2).

If the Chinese Healthier Choice Logo was adopted, the proportion of Chinese processed meat products that met the threshold for total fat was 4.9% to 69.1%; the proportion of French smoked and roasted meat products (3.3%) that met the threshold for total fat was the lowest. The proportion of processed meat products in China, the UK, and France that met the threshold for sodium was 4.4% to 77.8%. Between 34.8% and 50.8% of sausage products in the UK and France met the threshold for saturated fat. A large proportion of processed meat products in the UK and France met the threshold for total sugar, ranging from 99.7% to 100.0% (Supplementary Table S3).

## 4. Discussion

Processed meat products are pre-packaged foods commonly consumed in China [11]. As group 1 carcinogens, they have been demonstrated to be positively correlated with the risk of NCDs [15,16,17,18,19]. This study adopted a more meticulous approach in classifying processed meat products in the China Standardized Database for the Composition of Pre-Packaged Food and analyzed their nutrient content. Compared with the published articles, this study also analyzed the nutrient content of processed meat products in the National Open Database of the UK and France. Two NP models were used to compare the nutrient content of processed meat products from the three countries. This study provided information on the healthiness of Chinese processed meat products.

In this study, we observed that Chinese products were relatively low in energy and total fat but relatively high in protein and carbohydrates compared to smoked and roasted meat products in the UK and France. The sodium content of products in the UK was the highest at 1368 mg/100 g. In terms of sausage products, Chinese products were lower in energy, protein, and total fat, but higher in carbohydrates and sodium, compared with the UK and France. This result is consistent with a previous study conducted by Song et al. [20] on processed meat and fish products in five countries. In the bacon category, products in the UK had the highest sodium content at 1612 mg/100 g. In terms of sausage and hot dogs, Chinese products had the highest sodium content. The aforementioned study also found that out of all processed meat products combined, Chinese products had the highest sodium content. However, Chinese processed meat products in categories such as frozen meat and sausages and hot dogs had a lower sodium content than other countries [20]. These results indicate that a more specific classification of processed meat products would facilitate our understanding of the nutrient content of processed meat products and help the meat industry to reformulate processed meat products to reduce excessive intakes of CNs such as sodium, saturated fat and sugar.

Processed meat products of the three countries were also evaluated against the Chilean FOPWL and the Chinese Healthier Choice Logo criteria. The results showed that based on the Chilean FOPWL, only 1.1% of Chinese smoked and roasted meat products were compliant, and all products in other categories did not meet the criteria of this model. Between 53.7% and 100.0% of processed meat products from China, the UK, and France were identified as having excessive amounts of CNs in at least one category according to the Chinese Healthier Choice Logo criteria. Other research results showed that when using the Health Star Rating NP model, the mean HSR of meat and meat alternatives for 12 countries was 2.49, and for China, it was 1.89, 77% lower than India, which had the best average quality [39]. Under the Chilean NP model, the Pan American Health Organization (PAHO) NP model, and the WHO NP model for the Western Pacific Region, the proportion of meat and meat products included in FoodSwitch China data in 2017–2020 with excessive CNs was 95.3%, 99.3%, and 84.2%, respectively [38].

This study found that dried meat products contributed 75% of the sodium of the NRV per 100 g, while cured products contributed 64% of the total fat and 103.8% of the sodium. Jinhua ham, sauce pickled meat jerky, and Chinese bacon are Chinese ethnic meat products [40] which are popular among Chinese consumers with a high market share, but with a high sodium content. Processed meat products are the main category targeted for salt reduction in China. However, Song et al. [20] found that processed meat and fish products rarely meet the reduction targets. It may be related to the role of sodium played in maintaining the quality and safety of meat products, particularly in terms of seasoning, flavor enhancement, water retention, color enhancement, antibacterial effects, and preservatives [13]. Current techniques for reducing sodium in processed meat products include salt removal, salt replacers, functional modification, flavor modification, and physical modification [13,41,42]. Novel technological treatments such as ultrasound technology and high hydrostatic pressure may effectively reduce salt in meat products, and a combination of different tools may achieve the desired effect [42]. Therefore, technical issues should not become an obstacle to the high sodium content of processed meat products.

In Chile, if the content of CNs associated with NCDs exceeded predetermined criteria, the black octagonal FOPWL must be displayed on the front of the product packaging [35,36]. Schnettler et al. [43,44] assessed the influence of consumers’ perceived healthfulness, purchase intention, and willingness to pay for reformulated frankfurters. The results showed that sodium and fat reduction caused an increase in willingness to pay, and reformulated products without an FOPWL for sodium and saturated fat were perceived as healthier, leading to higher purchase intention scores. These results indicate that implementing FOPL in processed meat products can encourage food manufacturers to reformulate products.

It is noteworthy that China has made many efforts to reduce the content of CNs in processed meat products. For example, the Chinese Healthier Choice Logo has set different thresholds of low-level total fat, saturated fat, total sugar, added sugar, and sodium for 10 categories and 31 subcategories, including meats and products [25]. The Guidelines for Salt Reduction in Chinese Food Industry jointly issued by the NINH, the China CDC, and the Chinese Nutrition Society proposed targets for 2025 and 2030 for the step-wise reduction in salt in 16 categories and 16 subcategories, including 2 categories and 7 subcategories of processed meat products, which helps food manufacturers of processed meat products to develop priority strategies and key product salt reduction targets based on product characteristics, and encourages the food industry to increase investment in research and development of low-salt meat products to gradually reduce the proportion of high-salt foods in processed meat products [45]. In addition, the Chinese Nutrition Society, the NINH, and the China CDC are collaborating with the United Nations International Children’s Emergency Fund to develop NP models that suit the Chinese food environment and can effectively prevent and control diet-related NCDs. The FOPWL strategy and Nutri-Score have attracted the highest attention from this research team, as they can encourage consumers to purchase healthier products [24,46,47,48].

The strength of this study is that it included a large number of Chinese processed meat products, whose classification followed Chinese food standards, as well as the nutrient characteristics, processing technologies, formulation of processed meat products, and Chinese dietary habits. Compared with the published articles, this study analyzed the nutrient content of processed meat products in the National Open Database of the UK and France, and two NP models were used to compare the nutrient content of processed meat products from the three countries. An important limitation of the current research is that it was based on the nutritional information provided on the food label and therefore did not involve actual measurements of the nutrient content in processed meat products.

## 5. Conclusions

This study analyzed the nutrient content of seven categories of Chinese processed meat products, and smoked and roasted meat products and sausage products in the UK and France. Cured meat products had the highest median energy, total fat content, and sodium content; dried meat products had the highest median protein content and carbohydrate content. There were significant differences in energy content and contents of total fat, protein, and carbohydrates across different products in the three countries (*p* < 0.001). A large number of processed meat products currently collected in China, the UK, and France did not meet the criteria of the Chilean FOPWL and the Chinese Healthier Choice Logo. This study provided data to support the assessment of dietary intake of processed meat products at the population level and evidence of best practice to scientifically guide meat intake in China.

## Figures and Tables

**Table 1:** Nutrient and thresholds of the Chilean FOPWL and the Chinese Healthier Choice Logo.

NP Models	Food Category	Energy	Total Fat	Saturated Fat	Total Sugar	Sodium/Salt
Chilean FOPWL	Solid foods	≥275 kcal/100 g		≥4 g/100 g	≥10 g/100 g	≥400 mg/100 g
Chinese Healthier Choice Logo	Sausage products and canned meat products;		≤10 g/100 g	≤5 g/100 g	≤5 g/100 g	≤800 mg/100 g
	other processed meat products		≤10 g/100 g		≤5 g/100 g	≤800 mg/100 g

**Table 2:** Nutrient distribution of Chinese processed meat products.

Food Category	*n*	Energy (kcal/100 g)			Protein (g/100 g)			Total Fat (g/100 g)			Carbohydrate (g/100 g)			Sodium (mg/100 g)			Total Sugar (g/100 g) ^a^	Saturated Fat (g/100 g) ^b^
		Median	25th	75th	Median	25th	75th	Median	25th	75th	Median	25th	75th	Median	25th	75th	Median	Median
Cured meat products	41	483	332	545	18.3	15.4	22.2	38.7	23.3	46.6	15.6	5.1	19.2	2076	1580	3064	4.2	5.7
Prepared meat products	54	135	115	212	13.5	11.5	16.4	7.6	2.7	13.8	5.2	2.1	9.9	648	466	790	4.2	5.7
Soy sauce and pot-roast meat products	984	202	161	262	25.2	20.0	30.7	8.8	6.0	13.1	2.8	0.6	7.3	1240	967	1586	4.2	5.7
Dried meat products	457	366	341	380	30.2	26.1	34.2	8.6	6.3	10.7	38.2	31.3	43.0	1500	1239	1760	4.2	5.7
Smoked and roasted meat products	93	236	205	299	26.0	20.3	31.0	10.2	7.7	14.8	4.8	2.5	13.2	1150	883	1538	4.2	5.7
Canned meat products	62	257	231	314	11.2	10.0	13.0	19.7	14.8	24.5	5.5	2.7	8.2	795	719	858	4.2	5.7
Sausage products	195	187	164	213	13.0	11.6	16.0	10.9	8.5	13.0	9.2	6.0	12.3	980	826	1100	4.2	5.7
*p* value *		<0.001			<0.001			<0.001			<0.001			<0.001			NA	NA

Notes: * The Kruskal–Wallis H test was used to determine *p* values; statistical significance was indicated at *p* < 0.05. NA—not applicable. ^a,b^ The Standard on Nutrition Labelling of Pre-Packaged Foods (GB 28050-2011) specifies mandatory rules for nutrition labeling by manufacturers to provide quantitative information on energy, protein, fat, carbohydrate, and sodium content of foods and their contributions to the nutrient reference value (NRV). Thus, we derived the total sugar content and saturated fat content of processed meat products from the China Food Composition Tables Standard Edition [37] and the literature [38].

**Table 3:** Percentage of the nutrient content of Chinese processed meat products’ NRV *.

Food Category	Energy (%)			Protein (%)			Total Fat (%)			Carbohydrate (%)			Sodium (%)		
	Median	25th	75th	Median	25th	75th	Median	25th	75th	Median	25th	75th	Median	25th	75th
Cured meat products	24.2	16.6	27.3	30.5	25.7	37.0	64.5	38.8	77.7	5.2	1.7	6.4	103.8	79.0	153.2
Prepared meat products	6.8	5.8	10.6	22.5	19.2	27.3	12.7	4.5	23.0	1.7	0.7	3.3	32.4	23.3	39.5
Soy sauce and pot-roast meat products	10.1	8.1	13.1	41.8	33.3	51.2	14.7	10.0	21.8	0.9	0.2	2.4	62.0	48.4	79.3
Dried meat products	18.3	17.1	19.0	50.3	43.5	57.0	14.3	10.5	17.8	12.7	10.4	14.3	75.0	62.0	88.0
Smoked and roasted meat products	11.8	10.3	15.0	43.3	33.8	51.7	17.0	12.8	24.7	1.6	0.8	4.4	57.5	44.2	76.9
Canned meat products	12.9	11.6	15.7	18.7	16.7	21.7	32.8	24.7	40.8	1.8	0.9	2.7	39.8	36.0	42.9
Sausage products	9.4	8.2	10.7	21.7	19.3	26.7	18.2	14.2	21.7	3.1	2.0	4.1	49.0	41.3	55.0

Notes: * NRV of each nutrient: energy, 8400 kJ; protein, 60 g; total fat, 60 g; carbohydrate, 300 g; sodium, 2000 mg [27].

**Table 4:** Nutrient distribution of processed meat products in the UK and France.

Country	Food Category	*n*	Energy (kcal/100 g)			Protein (g/100 g)			Total Fat (g/100 g)			Carbohydrate (g/100 g)			Sodium (mg/100 g)			Total Sugar (g/100 g)			Saturated Fat (g/100 g)		
			Median	25th	75th	Median	25th	75th	Median	25th	75th	Median	25th	75th	Median	25th	75th	Median	25th	75th	Median	25th	75th
the UK	Smoked and roasted meat products	21	287	228	−324	23.8	17.7	25.3	21.6	16.2	25.1	0.0	0.0	0.0	1368	1073	1531	0.0	0.0	0.0	8.1	6.1	9.0
	Sausage products	23	267	191	−312	15.8	13.9	18.4	19.5	9.4	24.8	7.2	1.1	9.1	630	492	866	1.3	0.5	2.3	7.6	2.5	8.8
France	Smoked and roasted meat products	90	253	235	−266	17.4	17.0	18.2	20.0	18.1	22.0	0.8	0.8	1.0	1000	980	1200	0.8	0.7	1.0	7.7	6.7	8.8
	Sausage products	1023	230	117	−318	21.0	19.9	27.0	12.5	3.2	28.0	1.0	0.8	1.8	960	748	1800	0.9	0.5	1.3	4.8	1.2	11.0

## Data Availability

Data sets generated during the study are available from the corresponding author on reasonable request.

## Supplementary Materials

The following supporting information can be downloaded at: https://www.mdpi.com/article/10.3390/nu16050578/s1, Table S1. Classification and examples of main processed meat products in China. Figure S1; Selection process of processed meat products in the UK and France; Table S2. Number and proportion of processed meat products meeting the criteria of the Chilean FOPWL; Table S3. Number and proportion of processed meat products meeting the criteria of Chinese Healthier Choice Logo.
