# Supplementary material for: Evaluation of the Nutritional Quality of Chinese Processed Meat Products: Comparison of Two Nutrient Profile Models
Source: Nutrients. 2024 Feb 20;16(5):578. doi: 10.3390/nu16050578 (PMC10935351; doi:10.3390/nu16050578)
Supplement: Supplementary file 1 [file nutrients-16-00578-s001.zip › nutrients-2837520-supplementary.pdf]

Supplementary Materials:

Table S1. Classification and examples of main processed meat products in China.

Food category	Examples of Food Items	Type of standards	Standard
Cured meat products	Sausage of Guang flavor, Pickled meat in salt, etc	national standards, health standards	The Standard on Nutrition Labelling of Prepackaged Foods (GB 28050-2011), The Standards for Uses of Food Additive (GB 2760-2014), Regulation of Food Composition Data Expression (WS/T 464-2015), Cured Meat Products (GB 2730-2015)
Prepared meat products	Popcorn chicken, Beef steak, Mutton kebabs, Beef kebabs, Chicken nugget, Chicken steak, Chicken pieces, etc	national standards, health standards, group standards	The Standard on Nutrition Labelling of Prepackaged Foods (GB 28050-2011), The Standards for Uses of Food Additive (GB 2760-2014), Regulation of Food Composition Data Expression (WS/T 464-2015), Prefabricated Seasoned Meat Products (T/FJSP 0006-2020)
Soy sauce and pot-roast meat products	Meat in sauce, Plain boiled pork,<br>Pork ear in sauce, Pork feet in sauce, Chicken feet in sauce, Chicken wings in sauce,<br>Chicken drumsticks in sauce,<br>Duck wings in sauce, Beef in sauce, etc	national standards, health standards,	The Standard on Nutrition Labelling of Prepackaged Foods (GB 28050-2011), The Standards for Uses of Food Additive (GB 2760-2014), Regulation of Food Composition Data Expression (WS/T 464-2015), Soy Sauce and Pot-roast Meat Products (GB/T 23586-2009)
Dried meat products	dried meat floss, dried meat dice, dried meat slice, etc	national standards, health standards	The Standard on Nutrition Labelling of Prepackaged Foods (GB 28050-2011), The Standards for Uses of Food Additive (GB 2760-2014), Regulation of Food Composition Data Expression (WS/T 464-2015), Dried Meat Floss (GB/T 23968-2009), Dried Meat Dice (GB/T 23969-2009), Dried Meat Slice (GB/T 31406-2015)
Smoked and roasted meat products	Peking Roasted Duck, Chinese bacon, Bacon, Roasted chicken wings, Roasted Chicken drumsticks,	national standards, health standards	The Standard on Nutrition Labelling of Prepackaged Foods (GB 28050-2011), The Standards for Uses of Food Additive (GB 2760-2014), Regulation of Food Composition Data Expression

	Roasted Chicken neck, Roasted beef, etc		(WS/T 464-2015), Bacon (GB/T 23492-2009)
Canned meat products	Canned pork, Canned beef, Canned luncheon meat, etc	national standards, health standards, industry standards	The Standard on Nutrition Labelling of Prepackaged Foods (GB 28050-2011), The Standards for Uses of Food Additive (GB 2760-2014), Regulation of Food Composition Data Expression (WS/T 464-2015), Canned Stewed Beef (QB/T 1363-1991), Canned Steamed Beef (GB/T 13514-1992), Canned Pork in Natural Juice (GB/T 13513-1992), Canned Steamed Pork (QB/T 2786-2006)
Sausage products	Jinhua ham, Xuanwei ham, Cooked crued ham	national standards, health standards	The Standard on Nutrition Labelling of Prepackaged Foods (GB 28050-2011), The Standards for Uses of Food Additive (GB 2760-2014), Regulation of Food Composition Data Expression (WS/T 464-2015), Cooked Crued Ham (GB/T 20711-2006), Ham Sausage (GB/T 20712-2006)

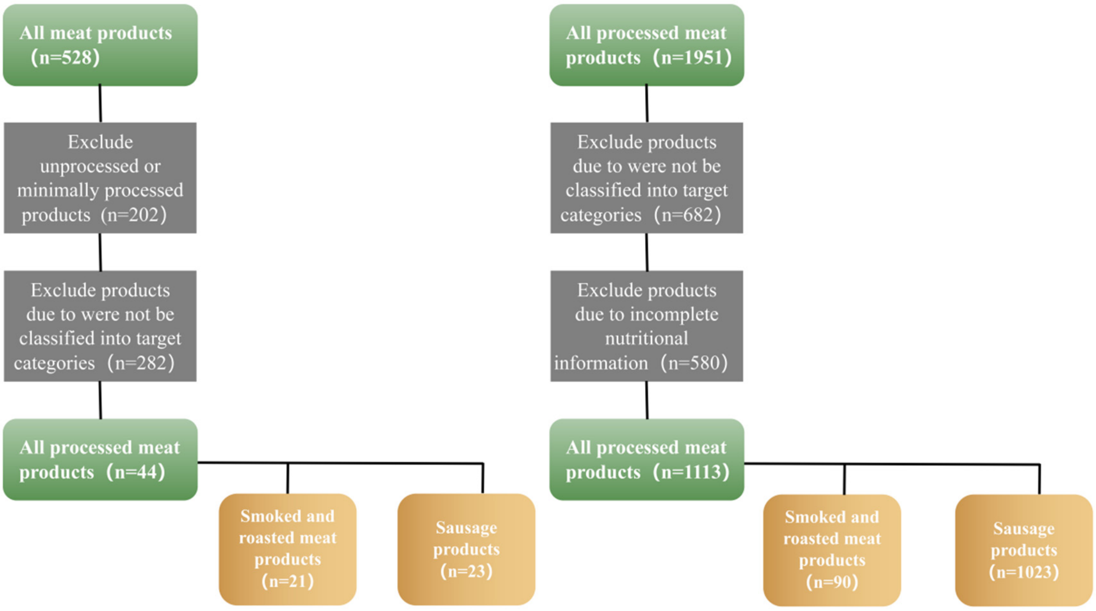

Figure S1. Selection process of processed meat products in the UK and France.

Table S2. Number and proportion of processed meat products meeting the criteria of the Chilean FOPWL.

Food category	Meets Criteria for Chilean FOPWL							
	Energy		Saturated fat		Total sugar		Sodium	
	n	%	n	%	n	%	n	%
Chinese cured meat products	2	4.9	-*	-	-	-	0	0.0
Chinese prepared meat products	53	98.1	-	-	-	-	9	16.7
Chinese soy sauce and pot-roast meat products	781	79.4	-	-	-	-	21	2.1
Chinese dried meat products	24	5.3	-	-	-	-	2	0.4
Chinese smoked and roasted meat products	67	72.0	-	-	-	-	1	1.1
Chinese canned meat products	36	58.1	-	-	-	-	0	0.0
Chinese sausage products	169	86.7	-	-	-	-	0	0.0
Smoked and roasted meat products in the UK	8	38.1	2	9.5	21	100.0	0	0.0
Sausage products in the UK	14	60.9	7	30.4	23	100.0	1	4.3
French smoked and roasted meat products	71	78.9	6	6.7	90	100.0	0	0.0
French sausage products	658	64.3	468	45.7	1022	99.9	1	0.1

Notes: * The Standard on Nutrition Labelling of Pre-packaged Foods (GB 28050-2011) specifies mandatory rules for nutrition labeling by manufacturers to provide quantitative information on energy, protein, fat, carbohydrate, and sodium content of foods and their contributions to NRV. Thus, we derived the the total sugar content and saturated fat content of processed meat products from *China Food Composition Tables Standard Edition* and literature .Therefore, this study did not evaluate the number and proportion of sugar and saturated fat in Chinese processed meat products that met the criteria of the Chilean FOPWL.

Table S3. Number and proportion of processed meat products meeting the criteria of Chinese healthier choice logo.

**Food category**	**Meets Criteria for Chinese healthier choice logo**							
	**Total fat**		**Saturated fat**		**Total sugar**		**Sodium**	
	**n**	**%**	**n**	**%**	**n**	**%**	**n**	**%**
Chinese cured meat products	2	4.9	-*	-	-	-	2	4.9
Chinese prepared meat products	34	63.0	-	-	-	-	42	77.8
Chinese soy sauce and pot-roast meat products	592	60.2	-	-	-	-	126	12.8
Chinese dried meat products	316	69.1	-	-	-	-	29	6.3
Chinese smoked and roasted meat products	44	47.3	-	-	-	-	13	14.0
Chinese canned meat products	11	17.7	-	-	-	-	31	50.0
Chinese sausage products	75	38.5	-	-	-	-	39	20.0
Smoked and roasted meat products in the UK	2	9.5	NA	NA	23	100.0	2	9.5
Sausage products in the UK	7	30.4	8	34.8	23	100.0	17	73.9
French smoked and roasted meat products	3	3.3	NA	NA	90	100.0	4	4.4
French sausage products	461	45.1	520	50.8	1020	99.7	324	31.7

Notes: * The Standard on Nutrition Labelling of Pre-packaged Foods (GB 28050-2011) specifies mandatory rules for nutrition labeling by manufacturers to provide quantitative information on energy, protein, fat, carbohydrate, and sodium content of foods and their contributions to NRV. Thus, we derived the the total sugar content and saturated fat content of processed meat products from *China Food Composition Tables Standard Edition* and literature .Therefore, this study did not evaluate the number and proportion of sugar and saturated fat in Chinese processed meat products that met the criteria of the Chinese Healthier Choice logo.
